# Serum high mobility group box-1 (HMGB1) is closely associated with the clinical and pathologic features of gastric cancer

**DOI:** 10.1186/1479-5876-7-38

**Published:** 2009-05-28

**Authors:** Hye Won Chung, Sang-Guk Lee, Heejung Kim, Duck Jin Hong, Jae Bock Chung, David Stroncek, Jong-Baeck Lim

**Affiliations:** 1 Department of Internal Medicine, Institute of Gastroenterology, Yonsei University College of Medicine, Seoul, Korea; 2 Department of Laboratory Medicine, Yonsei University College of Medicine, Seoul, Korea; 3 Department of Transfusion Medicine, Warren G Magnuson Clinical Center, National Institutes of Health, Bethesda, Maryland, USA

## Abstract

**Background:**

High mobility group box-1 (HMGB1) is a newly recognized factor regulating cancer cell tumorigenesis, expansion and invasion. We investigated the correlation between the serum HMGB1 levels and the clinical and pathologic features of gastric cancer and evaluated the validity of HMGB1 as a potential biomarker for the early diagnosis of gastric cancer.

**Methods:**

A total of 227 subjects were classified into 5 disease groups according to the 'gastritis-dysplasia-carcinoma' sequence of gastric carcinogenesis and their serum levels of HMGB1 were analyzed by an enzyme-linked immunosorbent assay (ELISA) method. Clinical parameters, International Union Against Cancer (UICC) TNM stage, cancer size, differentiation or lymphatic invasion, vascular or perineural invasion and prognosis were used as analysis variables.

**Results:**

The serum HMGB1 levels were significantly different among disease groups (ANOVA, *p < 0.05*) and HMGB1 levels tended to increase according to the progression of gastric carcinogenesis. Serum HMGB1 levels were significantly associated with depth of invasion, lymph node metastasis, tumor size, and poor prognosis (*p < 0.05*). However, HMGB1 levels were not associated with patient gender or age, differentiation of tumor cells, or lymphatic, vascular and perineural invasion, or the existence of distant metastasis in advanced cancer (*p > 0.05*). The sensitivity and specificity of serum HMGB1 was 71% and 67% (cut-off value of 5 ng/ml) for the diagnosis of early gastric cancer, and 70% and 64% (cut-off value of 4 ng/ml) for the diagnosis of high-risk lesions, respectively. These values were greater than those for carcinoembryonic antigen (CEA) (30–40% of sensitivity).

**Conclusion:**

HMGB1 appears to be a useful serological biomarker for early diagnosis as well as evaluating the tumorigenesis, stage, and prognosis of gastric cancer.

## Background

A group of molecules that may act as mediators of angiogenesis are the so-called high-mobility group proteins. An important member of this superfamily is high mobility group box-1 (HMGB1) which was originally characterized as a non-histone, nuclear DNA-binding protein [1,2]. HMGB1 has been recently demonstrated to serve as a cytokine that mediates late lethal systemic inflammation via its extracellular release from activated macrophages/monocytes and cells undergoing necrosis [3-5]. The constant release of HMGB1, which functions as a proinflammatory cytokine, from necrotic tumor cells creates a microenvironment similar to chronic inflammation; a condition known to contribute to the development of epithelial malignancies, particularly inflammation-associated cancer [6]. In fact, many previous studies have demonstrated the over-expression of HMGB1 with its receptor, receptor for advanced glycation end products (RAGE), in different tumor types, including breast carcinoma [7], colorectal cancer [8], prostate cancer [9], pancreatic cancer [10], and hepatocellular carcinoma [11]. Moreover, these studies showed that the over-expression of HMGB1 is strongly correlated with tumor invasiveness [7-13].

Multiple steps and multiple factors are involved in the development of gastric cancer (GC). Among these factors, chronic inflammation is important particularly in the intestinal type of GC. The Correa hypothesis postulates that a progression from chronic gastritis to gastric atrophy, intestinal metaplasia (IM), dysplasia, and finally to cancer ('gastritis-dysplasia-carcinoma' sequence) [14]. In each step of GC progression many cytokines and intracellular signaling are involved [14].

Several studies have demonstrated that HMGB1 is over-expressed in approximately 85% of GC [15]. In addition, the over-expression of HMGB1 in GC is reported to be associated with tumor invasiveness and metastasis [15-17]. In almost all of these studies, the over-expression of HMGB1 has been documented in tissues by measuring mRNA levels via in situ hybridization or immunohistochemical analysis [7-10,15-17], but there is little information about the corresponding serological activity of HMGB1 and the progression of GC. Although the measurement of HMGB1 activity in tissues is clinically important, this method of biomarker analysis is somewhat limited because the measurement of biomarker activity in tissue requires invasive techniques such as endoscopy and biopsy, that are associated with patient discomfort and risk. HMGB1 could be measured in serum and used as a serologic tumor biomarker because it can be released into extracellular environment like other cytokines [6,11].

Although the overall incidence of GC has decreased in most countries over the past few decades, it is still a serious health problem [18]. The prognosis of advanced gastric cancer (AGC) with extensive node invasion and metastasis remains poor while early gastric cancer (EGC) is associated with excellent long-term survival [19]. Therefore, efforts to identify a serum biomarker that could be used to detect early stage GC or premalignant lesions as well as to estimate tumor invasion and predict prognosis are of great clinical importance. Although carcinoembryonic antigen (CEA) is a well-known tumor marker of GC, it is considered to be neither sensitive nor specific for GC screening [20,21].

In this study we measured serum HMGB1 and CEA levels and evaluated the correlation of these values with the progression of gastric carcinogenesis. We then estimated the validity of HMGB1 as a potential biomarker for the screening, diagnosis, and surveillance of GC. We also analyzed the relationship between serum HMGB1 levels and the clinical and pathological parameters of GC.

## Methods

### Subjects

Between March 2007 and July 2008 a total of 227 subjects were enrolled in this study at Severance Hospital, Gastroenterology Department Clinics, Yonsei University Health System. All subjects underwent upper gastrointestinal endoscopy (Types XQ-260, Olympus, Tokyo, Japan) with a biopsy. The final diagnosis was made by histopathological studies; via biopsy specimens in the non-cancer groups and via biopsy and surgical specimens in the cancer groups. All cancer patients were diagnosed for the first time during the enrollment period and their blood samples were collected before they received any treatment such as surgery, chemotherapy or radiotherapy. This research was approved by the Institutional Review Board of Yonsei University Health System and all participants gave written informed consent.

Subjects were classified into 5 groups based on endoscopic biopsy findings according to the 'gastritis-dysplasia-carcinoma' sequence in gastric carcinogenesis [14]; normal group (including acute and chronic gastritis (CSG), erosion, and gastric ulcer), high-risk group (including IM and adenoma), EGC group, AGC group without distant metastasis, and metastatic GC group including carcinomatosis (M group). All subjects were both age- and sex-matched among disease groups.

Subjects were excluded if they suffered from acute infection or inflammatory disease. They were also excluded if they had a history of chronic illness such as: autoimmune disease, rheumatic disease, chronic infection or inflammatory disease, or other cancers. Subjects with a history of previous gastric surgery or any other treatment for GC such as chemotherapy or radiotherapy were also excluded.

In the cancer groups all patients were evaluated by imagining procedures such as chest X-ray, helical computed tomography (CT), and whole body Positron Emission Tomography (PET) scan. This was followed by gastrectomy with lymph node dissection.

### Measurement of serum CEA and HMGB1 levels

Approximately 10 ml of whole blood was collected in non-heparinized tubes from each fasting subject and allowed to clot at room temperature for half an hour. The blood was centrifuged at 3,000 rpm for 15 minutes and the serum fraction was aliquoted and stored at -70°C in microfuge tubes until assayed. CEA was measured with a Roche E170 (Roche Diagnostics GmbH, Mannheim, Germany), a modular immunoassay analyzer. HMGB1 was measured by the commercially available HMGB1 ELISA Kit II (SHINO-TEST Corporations, Kanagawa, Japan). Briefly, 100 μl of sample diluent was added to each well and then 10 μl of standard, and sample or control was added to the well. The microtiter plates were incubated for 20–24 h at 37°C. After washing, 100 μl/well of anti-human HMGB1 peroxidase-conjugated monoclonal antibody was added and the plates were incubated at room temperature for 2 h. After washing, the chromogen 3,3',5,5'-tetra-methylbenzidine was added to each well. The enzyme reaction was allowed to proceed for 30 min at room temperature. The chromogenic substrate reaction was stopped by the addition of stop solution (0.35 mol/l Na_2_SO_4_) and the absorbance was read at 450 nm. The results were calculated using a calibration curve prepared from standards.

### Histopathological analysis

In non-cancer groups two specimens were obtained from the greater curvature of the antrum and the midpoint of the greater curvature of the gastric body via endoscopic biopsy. In the patients with cancer two specimens were obtained from the cancerous portion of the surgical specimen and adjacent normal mucosa. All formalin-fixed and paraffin-embedded specimens were stained with 0.7% Harris hematoxylin solution (w/v) (Sigma, Missouri, USA) to confirm the pathology and stained with Giemsa solution (Sigma, Missouri, USA) to detect *H. pylori *infection. Glandular atrophy and IM were diagnosed according to the updated Sydney classification [22]. With surgical specimens of GC, conventional pathological parameters of GC (tumor size, tumor location, metastatic distant organ, depth of invasion, lymph node metastasis, and lymphovascular and perineural invasion) were analyzed. Pathological differentiations were classified using Lauren classification. Stages were analyzed according to the International Union Against Cancer (UICC)-TNM stage [23]. Two pathologists who were blind to previous histological scores and other experimental results determined the histopathological results.

### Statistical analysis

Values (CEA, HMGB1) in each group were expressed as a mean with the 25–75% standard deviation range. One-way analysis of variance (ANOVA) with the multiple comparisons by Post HOC Scheffe method was used to compare the mean of each value (CEA, HMGB1) among groups. Pearson correlation analysis was performed to assess the correlations between HMGB1 and the continuous variables, and Spearman correlation was performed to assess the correlations between HMGB1 and the non-continuous variables. Receiver operating characteristic curves (ROC) were plotted to determine the best cut-off ranges for GC screening for each value, and the relevant sensitivities and specificities were calculated. Survival time was measured in days from the day of first diagnosis to death or last review in M group. Overall survival times were analyzed by the Kaplan-Meier method. Statistical Package for Social Sciences software (SPSS, Chicago, Illinois, USA version 13.0) was used for data support and analysis and p-values < 0.05 were considered as statistically significant differences.

## Results

### Characteristics of the subjects

The 227 subjects studied included 50 patients with normal gastric mucosa including chronic gastritis and ulcer (normal group), 50 with IM including adenoma (high risk group), 40 with EGC (EGC group), 45 with AGC without distant metastasis (AGC group), and 42 with metastatic GC (M group). The clinical and pathological characteristics in each subject and their tumor are described in Table 1. There were no significant differences among 5 disease groups in demographics such as age, gender, and the status of *H. pylori *infection (ANOVA, *p > 0.05*).

**Table 1:** Baseline clinico-pathologic characteristics and serum level of CEA or HMGB1 according to disease groups

Groups of diseases (n)	Normal (50)	High-risk Group (50)*	EGC Group (40)	AGC Group (45)	Metastatic GC group (42)
Clinical factors					
Age (mean ± S.D; year)	56.0 ± 13	57.5 ± 12.3	61.5 ± 12.0	59.8 ± 13.7	54.7 ± 11.9
Male/female (n)	32:18	31:19	25:15	28:17	26:16
*H. pylori *infection (-/+, n)	21:29	30:20	18:22	27:18	20:22
Pathological factors					
Size of main tumor (cm)	NS	NS	4.1 ± 2.7	9.2 ± 5.9	11.8 ± 4.6
Differentiation	NS	NS			
Intestinal type			25	21	14
Diffuse type			15	24	28
Tumor location	NS	NS			
Antrum/Body			31	28	21
Cardia			9	10	12
Diffuse			0	7	7
Depth of invasion	NS	NS			
m, sm			24, 16	2, 0	0, 0
mp, ss			0	9, 13	3, 10
se, a_1–3_			0	19, 2	1, 0
Lymph-node metastasis	NS	NS			
N0			39	9	1
N1			1	17	2
N2			0	10	3
N3			0	9	8
Lymphovascular invasion (-/+)	NS	NS	33: 7	10: 35	6: 10
Distant metastatic organ	NS	NS	NS	NS	
Liver					18
Peritoneum					19
Others^†^					16
Stage	NS	NS			
I			40		
II				19	
III				26	
IV					42
Serum CEA (ng/ml)^‡^	1.7 ± 0.8	2.6 ± 1.8	1.6 ± 0.9	3.8 ± 8.1	46.3 ± 551.5
Serum HMGB1 (ng/ml)^§^	3.9 ± 3.4	6.3 ± 6.3	9.9 ± 11.5	16.5 ± 27.4	14.1 ± 13.2

EGC, early gastric cancer; ACG, advanced gastric cancer; GC, gastric cancer; *H. pylori*, Helicobacter pylori; mucosa; sm, sub-mucosa; mp, muscularis propria; ss, sub-serosa; se, serosa, a1, adventitia; a2, definite invasion into adventitia; a3, invasion into neighboring structures; N0, no lymph node metastases; N1, 1 to 6 regional lymph node metastases; n2, 7 to 15; N3, greater than 15; NS, not studied.

*This group includes intestinal metaplasia and adenoma.

^† ^Others include ovary, pancreas, colon, bone, adrenal gland, lung, etc.

^‡^Serum CEA level was not significantly different among normal, high-risk, EGC, and AGC groups (ANOVA, *p *> *0.05*). It was merely significantly higher in metastatic GC group (M group) compared with other groups (*p *<*0.05*).

^§^Serum HMGB1 level was significantly difference among normal, high-risk, EGC, AGC, and M groups (ANOVA, *p *<*0.05*). And serum HMGB1 level tended to increase according to the progression of the gastric carcinogenesis.

^¶^Pathological factors such as depth of invasion, node metastasis, frequency of lymphovascular or perineural invasion were not fully investigated in M group because most of patients in this stage could not be received an operation.

### Serum levels of CEA and HMGB1 among disease groups

**Figure 1:**
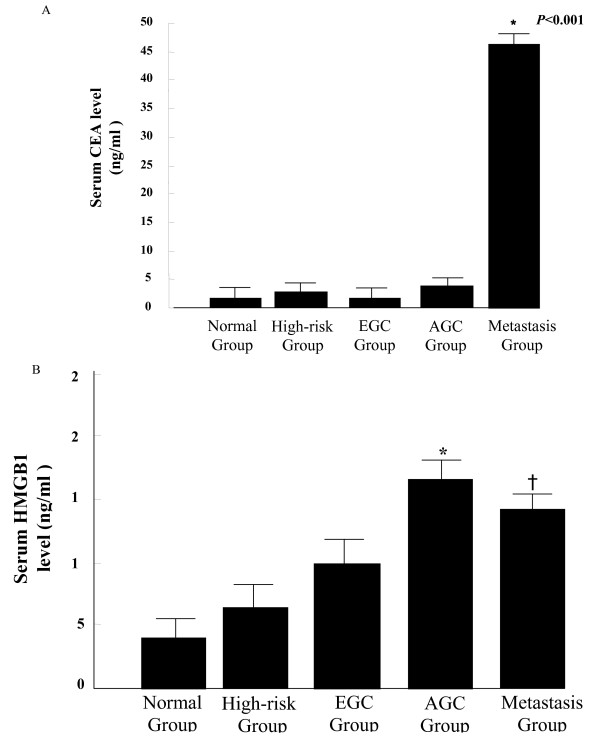
**Serum levels of CEA and HMGB1 according to disease group**. Figure 1A shows that the serum levels of CEA were not significantly different among disease groups except for the metastatic GC group (M group). The serum levels of CEA in M group (*) were significantly higher than those of the other groups (Fig 1A, ANOVA with Post HOC Scheffe, *p < 0.01*). Figure 1B showed that the serum levels of HMGB1 were significant different among all disease groups and tended to increase according to the progression of the gastric carcinogenesis (ANOVA with Post HOC Scheffe, *p < 0.05*). However, HMGB1 levels were not significantly different between the AGC group (*) and M group (†) (ANOVA with Post HOC Scheffe, *p > 0.05*). GC, gastric cancer; EGC, early gastric cancer; AGC, advanced gastric cancer.

The mean serum CEA and HMGB1 levels were compared among disease groups (Table 1 and Figure 1). The serum CEA levels were 1.7 ± 0.8 ng/ml in the normal group, 2.6 ± 1.8 ng/ml in the high-risk group, 1.6 ± 0.9 ng/ml in the EGC group, 3.8 ± 8.1 ng/ml in the AGC group, and 46.3 ± 551.5 ng/ml in M group. No significant differences in CEA levels were found among the normal, high-risk, EGC, and AGC groups (ANOVA, *p > 0.05*). Serum CEA levels were only significantly greater in M group compared with the other groups (ANOVA, *p < 0.05*). (Figure 1A).

The mean serum HMGB1 levels were 3.9 ± 3.4 ng/ml in the normal group, 6.3 ± 6.3 ng/ml in the high-risk group, 9.9 ± 11.5 ng/ml in the EGC group, 16.5 ± 27.4 ng/ml in the AGC group, and 14.1 ± 13.2 ng/ml in M group. The serum HMGB1 levels were significantly different among the each group (ANOVA, *p < 0.05*). The HMGB1 levels tended to increase according to the progression of gastric carcinogenesis. Serum HMGB1 levels in subjects with cancer were significantly higher than the levels in those without cancer (*p < 0.05*), and the HMGB1 levels were also significantly greater in subjects in high-risk group compared to the normal group (*p < 0.05*). In the normal group, the HMGB1 levels were slightly higher in ulcer group than non-ulcer group, but the difference was not statistically significant (*p > 0.05*) (Figure 1B).

### Relationship between serum CEA or HMGB1 levels and clinical andpathological characteristics

When all subjects were included in the analysis, there was no relationship between the serum CEA level and age (Pearson correlation coefficients, γ_p _= 0.095, *p > 0.05*) or gender (Spearman correlation coefficients, γ_s _= -0.044, *p > 0.05*) (Table 2). Serum HMGB1 levels were also not related to age (γ_p _= 0.095, *p > 0.05*), or gender (γ_s _= -0.106, *p > 0.05*) although HMGB1 levels were slightly higher in males (Table 2). Neither CEA nor HMGB1 levels were influenced by *H. pylori *infection in any of the disease groups (γ_s _= -0.046, *p > 0.05 *and γ_s _= -0.121, *p > 0.05*, respectively) (Table 2). In addition, there was no significant correlation between HMGB1 and CEA levels in any of the disease groups (Pearson correlation = 0.081, *p > 0.05*) (Table 2).

**Table 2:** Relationship between serum CEA and HMGB1 levels and clinical characteristics in all patients

Variables	CEA	*P-*value	HMGB1	*P-*value
Age* (γ_p_)	0.078	*p > 0.05*	- 0.044	*p > 0.05*
Sex^† ^(γ_s_)	0.071	*p > 0.05*	- 0.187	*p = 0.005*
H. pylori infection^† ^(γ_s_)	- 0.121	*p > 0.05*	- 0.046	*p > 0.05*
CEA*(γ_p_)	-	-	0.081	*p > 0.05*
HMGB1*(γ_p_)	0.081	*p > 0.05*	-	-

* This variable is evaluated by Pearson correlation (for continuous variables). γ_p_, Pearson correlation coefficients.

^† ^This variable is evaluated by Spearman correlation (for non-continuous variables). γ_s_, Spearman correlation coefficients.

### Correlation between serum CEA or HMGB1 levels and the pathological differentiation of GC

According to the Lauren classification system (intestinal vs. diffuse type), no significant difference in either CEA or HMGB1 levels was found among all differentiation groups; the Spearman correlation coefficients (γ_s_) were -0.040 (*p *> *0.05*) and -0.009 (*p *> *0.05*), respectively (Table 3).

**Table 3:** Relationship between serum CEA or HMGB1 level and pathological characteristics in gastric cancer groups including EGC, AGC, and metastatic GC

Variables (γ_s_)	CEA	*P*-value^¶^	HMGB1	*P*-value^¶^
Depth of invasion (T stage)	0.118	*p *> *0.05*	0.273	*p *= *0.011*
Lymph node metastases (N stage)	0.131	*p *> *0.05*	0.225	*p *= *0.039*
Pathological differentiation*	-0.040	*p *> *0.05*	0.009	*p *> *0.05*
Lymphovascular or perineural invasion	0.105	*p *> *0.05*	0.067,	*p *> *0.05*
Tumor location^†^	0.037	*p *> *0.05*	0.041	*p *> *0.05*
Size of tumor^‡^	0.147	*p *> *0.05*	0.457	*P *= *0.017*
Stage^§^	0.098	*p *> *0.05*	0.221	*p *= *0.040*

*Gastric cancers are divided into intestinal and diffuse type carcinoma according to Lauren classification of differentiation.

^†^Gastric cancers are divided into three groups according to tumor location; antrum/body, cardia, diffuse.

^‡^Gastric cancers are divided into three groups by size; < 3 cm, 3–5 cm and > 5 cm.

^§^The stage of tumor was defined according to the UICC-TNM classification. Subjects with stage IV were excluded from this analysis.

^¶ ^The relationship between each pathological variable and serum CEA or HMGB1 level in gastric cancer groups including EGC, AGC, and metastatic GC was evaluated by Spearman correlation (for non-continuous variables). γ_s_, Spearman correlation coefficients.

Tumor location had no effect on HMGB1 (ANOVA, *p > 0.05*) or CEA levels (ANOVA, *p > 0.05*) (Table 3), and in GC patients with metastatic lesions (M group), there was no difference in serum HMGB1 levels (ANOVA, *p > 0.05*) or CEA levels (ANOVA, *p > 0.05*) (Table 3) according to distant metastatic organs.

We divided the patients with measurable GC into three groups by primary tumor size: < 3 cm; 3–5 cm and > 5 cm in order to analyze the relationship between the serum HMGB1 levels and GC size (Table 3). There were significant differences among the three groups (ANOVA, *p < 0.05*, Table 1), and a significant positive correlation between HMGB1 level and tumor size (Spearman correlation coefficients, γ_s _= 0.457, *p < 0.05*). However, there was no significant correlation between CEA levels and tumor size (Spearman correlation coefficients, γ_s _= 0.147, *p > 0.05*).

### Relationship between the serum HMGB1 and CEA levels and GC TNM stage

HMGB1 levels were significantly correlated with depth of invasion [Spearman correlation coefficients (γ_s_) = 0.273 (*p < 0.05*)), and lymph node metastasis [Spearman correlation coefficients (γ_s_) = 0.225 (*p < 0.05*)) (Table 3), but CEA levels were not. However, serum HMGB1 levels were no higher in the M group than in the AGC group without distant metastasis. There was no significant correlation between HMGB1 levels and the frequency of lymphovascular or perineural invasion in any of the cancer groups (γ_s _= 0.067, *p > 0.05*). The HMGB1 level was significantly correlated with stage by UICC-TNM classification except for stage IV.

### Evaluation of the sensitivity and specificity of HMGB1 compared to CEA for the diagnosis of high-risk lesions and EGC

**Figure 2:**
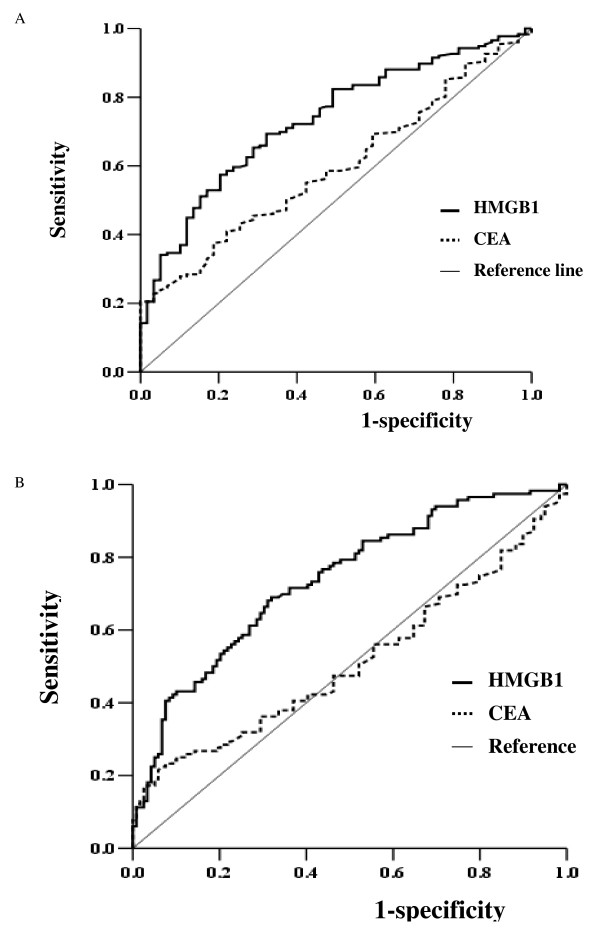
**Receiver operator characteristics (ROC) curves generated with serum CEA and HMGB1 levels for the detection of high-risk lesions (A) including intestinal metaplasia (IM) and adenoma and the detection of EGC (B)**. The figures indicate that serum HMGB1 levels demonstrated a higher sensitivity and specificity for the diagnosis of EGC and high-risk lesions of GC than CEA. GC, gastric cancer; EGC, early gastric cancer.

The CEA and HMGB1 cut-off points that gave the best sensitivity and specificity for the diagnosis of high-risk lesions (IM and adenoma) and cancer (EGC) were evaluated using area under the curve (AUC) analysis (Figure 2, Table 4 and Table 5). The sensitivity and specificity of serum CEA for the diagnosis of cancer (EGC) was 28% and 79% (cut-off value of 3 ng/ml), and 40% and 66% (cut-off value of 2.5 ng/ml), respectively. In contrast to CEA, the sensitivity and specificity of serum HMGB1 levels for the diagnosis of cancer (EGC) was 67% and 71% (cut-off value of 5.5 ng/ml), and 71% and 67% (cut-off value of 5 ng/ml). The sensitivity and specificity of serum CEA levels for the diagnosis of high-risk lesions was 39% and 76% (cut-off value of 2.5 ng/ml), and 49% and 62% (cut-off value of 2 ng/ml). In contrast to CEA, the sensitivity and specificity of serum HMGB1 levels for the diagnosis of high-risk lesions was 66% and 72% (cut-off value of 4.5 ng/ml), and 70% and 64% (cut-off value of 4 ng/ml). There was no difference in sensitivity and specificity of serum CEA and HMGB1 levels among the cancer groups with different histologic types (*p > 0.05*).

**Table 4:** Comparison of Cut-off Values, Sensitivity, and Specificity between serum CEA and HMGB1 levels for the screening of high-risk group (IM and adenoma)

	CEA (ng/ml)		HMGB1 (ng/ml)	
Cut-off value (ng/ml)	2.5	2	4.5	4

Sensitivity (%)	39	49	66	70
Specificity (%)	76	62	72	64

**Table 5:** Comparison of Cut-off Values, Sensitivity, and Specificity between serum CEA and HMGB1 levels for the screening of EGC group

	CEA (ng/ml)		HMGB1 (ng/ml)	
Cut-off value (ng/ml)	3	2.5	5.5	5

Sensitivity (%)	28	40	67	71
Specificity (%)	79	66	71	67

### Survival analysis GC in relation to serum HMGB1 levels

**Figure 3:**
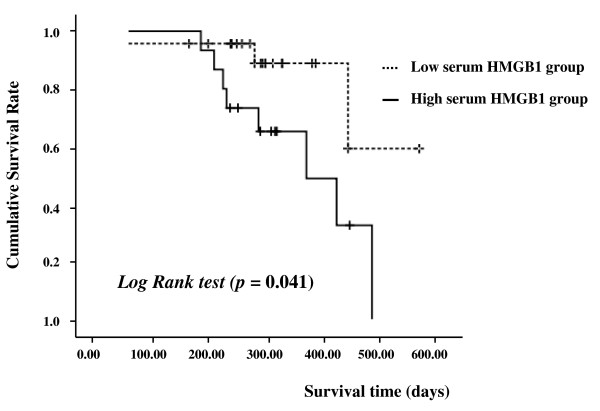
**Survival analysis of metastatic GC (M) group in relation to serum HMGB1 levels**. The prognosis of subjects in M group who had serum HMGB1 levels above the mean (> 14 ng/mL) was significantly poorer than those in M group with levels below the mean (Kaplan Meier method, Log-rank test, n = 42, *p *< 0.05).

We evaluated the relationship between the overall survival and the serum HMGB1 levels in selective metastatic GC patients (M group). The prognosis of those in the high HMGB1 level group defined as values above the mean HMGB1 level (> 14 units) was significantly poorer than for those in the low level group (values below the mean (Kaplan Meier method, Log-rank test, n = 42, *p *= 0.037, Figure 3).

## Discussion

In this study, we evaluated the validity of HMGB1 as a serological biomarker for GC and demonstrated for the first time that serum HMGB1 levels are significantly and sequentially increased in GC according to the progression of disease based on the theory of gastric carcinogenesis ('gastritis-dysplasia-carcinoma' sequence). Serum HMGB1 levels were significantly increased in patients with GC compared to those of patients without cancer. Although HMGB1 and its receptor, RAGE, are thought to be elevated in almost all types of cancer [6-13,15-17], recent studies have revealed that this is not true for all cases. For example, in lung cancer, the expression of HMGB1 and RAGE are reduced [24]. Therefore, individual studies of HMGB1 levels in each type of cancer are needed to obtain a more accurate understanding of HMGB1-related mechanisms in cancer development and progression.

Several studies have reported that HMGB1 was over-expressed in GC tissue, and the over-expression of HMGB1 plays an important role in the process of GC tumorigenesis, expansion, and invasion [15-17]. Quantification of HMGB1 expression in tissues could be clinically useful; however, the utility of the evaluation of HMGB1 over-expression in tissue is somewhat limited because of the relatively higher-risk, invasiveness and high cost of the procedures used to obtain the tissue: endoscopic biopsy or surgical resection.

A recent study found that HMGB1 could be detected in the serum of cancer patients because it can be passively released from dying tumor cells, or actively released from immune cells into the extra-cellular space or serum [1-5,11]. In the present study, we quantified serum HMGB1 levels in patients who had normal gastric mucosa, IM or adenoma and carcinoma. The serum levels of HMGB1 were increased sequentially according to GC disease stage based on the theory of gastric carcinogenesis and HMGB1 levels were significantly different between normal and high-risk lesions of GC as well as between cancer and non-cancer. These results are consistent with those of Kuniyasu *et al*. in which HMGB1 was measured in GC tissue. We did not divided the group of patients with high-risk lesions into an IM and adenoma group because the number of patients with adenoma was very small (n = 11) and this number of patients would not have had the statistical power to detect a difference in mean value between these two disease groups. However, serum HMGB1 levels tended to be elevated in patients with adenoma compared to patients with IM (data was not shown).

We also compared the sensitivity and specificity of serum HMGB1 with that of CEA, a well-known gastrointestinal tumor biomarker. Many other studies have shown that CEA has only 30–40% sensitivity for the detection of EGC or high-risk lesions and this is very similar to the results of our study [20,21]. However, the sensitivity and specificity of serum HMGB1 was about 71% and 67% (cut-off value of 5 ng/ml) for the detection of cancer (EGC), and 70% and 64% (cut-off value of 4 ng/ml) for the detection of high risk lesions (IM or adenoma). These results are very dramatic compared with previous studies of other GC biomarkers [20,21,25,26].

In our study, serum HMGB1 levels did not differ according to histologic type (intestinal vs. diffuse) and this is similar to the results by Kuniyasu *et al*. [15]. These results may mean that HMGB1 was not affected by tissue histologic type, and HMGB1 might be better than other biomarkers such as pepsinogen or gastrin which are important markers for the intestinal type of GC but not the diffuse type [25,26].

This study found that serum levels of HMGB1 were associated with depth of invasion (T stage), lymph node metastasis (N stage), tumor size, and poor prognosis. However, HMGB1 levels were not associated with patient gender, age, and lymphovascular or and perineural invasion. In addition, HMGB1 levels did not differ between AGC without distant metastasis and metastatic GC. For metastasis to occur, tumor cells must pass through a multi-step process involving a series of sequential and selective events [27,28]. Therefore, our results might be caused by the multiple factors in addition to HMGB1 that contribute to distant metastasis in contrast to tumor expansion and local invasion. These factors include a variety of other cytokines or signaling molecules.

In this study, we could not analyze the overall survival of all cancer patients. Those in the EGC and AGC groups without distant metastasis were excluded because the study period was too short to evaluate the survival of patients with EGC or resectable AGC. For this reason, we limited the evaluation of the relationship between the overall survival and the serum HMGB1 levels to patients in the metastatic GC group. Serum HMGB1 levels were inversely correlated with the overall survival time of patients with far-advanced stage of GC (Figure 3). In addition, HMGB1 levels were closely related with lymph node metastasis. In a previous study, lymph node metastasis was found to be an independent prognostic factor of GC [29]. Therefore, our data may imply that HMGB1 is associated with poor prognosis of GC. However, further studies to evaluate the exact relationship between HMGB1 levels and prognosis of GC are needed by including all GC patients and following-up for a long time enough to evaluate the overall survival time of all GC patients.

Because HMGB1 is over-expressed in GC, blocking of HMGB1 production or release, or preventing its interaction with its receptor(s) might provide an important opportunity for the prevention or treatment of GC as shown in a colitis-associated cancer model [30]. However, several studies have demonstrated that HMGB1 mediates endogenous Toll-like receptor (TLR) activation, and its increased interaction might enhance the tumor regression by immunoadjuvant effects after conventional chemo- or radiotherapy [31,32]. Therefore, further studies are needed to reach on a deeper understanding of the biology of HMGB1 in GC and to evaluate its therapeutic usefulness.

## Conclusion

HMGB1 is a new serologic biomarker for the screening, diagnosis, and surveillance of GC in high-incidence areas such as Korea.

## Competing interests

The authors declare that they have no competing interests.

## Authors' contributions

HWC, JBC, and JBL designed the study. HWC, SGL, DJH and HJK collected and stored all the samples. HWC and SGL acquired quantitative serum HMGB1 concentration data. JBL acquired quantitative serum CEA concentration data. HWC, SGL and DJH performed data analysis and histopathological correlations, JBL supervised all experiments. HWC, JBC, DS and JBL drafted and edited the manuscript. All authors read and approved the final manuscript.
